# Understanding how users identify health misinformation in short videos: an integrated analysis using PLS-SEM and fsQCA

**DOI:** 10.3389/fpubh.2025.1713794

**Published:** 2025-12-08

**Authors:** Ruojia Wang, Huiwen Yang, Yue Wang, Xing Zhai

**Affiliations:** 1 School of Management, Beijing University of Chinese Medicine, Beijing, China; 2 School of Traditional Chinese Medicine, Beijing University of Chinese Medicine, Beijing, China; 3 Centre for Evidence-Based Chinese Medicine, Beijing University of Chinese Medicine, Beijing, China

**Keywords:** health misinformation, short videos, influencing factors, PLS-SEM, fuzzy-set qualitative comparative analysis (fsQCA), health information governance

## Abstract

**Background:**

Short-video platforms have become major channels for public access to health information in the digital era. However, the low barriers to content creation and the increasing use of AI-generated content have accelerated the spread of health misinformation, underscoring the need to better understand how users identify health misinformation in short videos.

**Methods:**

Grounded theory was applied to analyze 47 in-depth interviews and extract core factors influencing users’ recognition of health misinformation in short videos. Based on the derived factor structure, a questionnaire survey was conducted and 279 valid samples were collected. Partial least squares structural equation modeling (PLS-SEM) was used to test the proposed relationships, and fuzzy-set qualitative comparative analysis (fsQCA) was further employed to identify the causal configurations through which different factor combinations contribute to users’ health misinformation discernment.

**Results:**

The results identified three key categories: information quality, user characteristics, and external environments. The PLS-SEM model demonstrated acceptable explanatory power (*R^2^* = 0.478) for users’ health misinformation discernment in short videos. Among the seven proposed hypotheses, content logic (*p* < 0.05), narrative expression (*p* < 0.05), information structure (*p* < 0.01), cognitive level (*p* < 0.05), and external influences (*p* < 0.05) were statistically supported, while information reliability and psychological needs showed non-significant effects. The fsQCA further revealed three distinct causal configurations leading to effective discernment. When content logic functioned as the core condition, users tended to rely on central, analytical processing; whereas when external influences were dominant, users were more likely to depend on heuristic processing rather than message logic.

**Discussion:**

The findings highlight three distinct ways users process health misinformation in short videos, including primarily analytical evaluation, peripheral reliance on content cues, and peripheral reliance on cognitive cues. These results suggest practical strategies for mitigating health misinformation on short-video platforms, emphasizing interventions at individual, platform, and policy levels.

## 1 Introduction

The rapid digitization of health communication has transformed social media platforms into major channels for public health information dissemination (1, 2). Among these platforms, short-video services such as TikTok, YouTube Shorts, and Douyin have become increasingly important sources of health-related information (3, 4). In China, over 1.07 billion people used short-video services by the end of 2024, accounting for 93.8% of all internet users (5). The visual storytelling style, concise format, and personalized algorithm recommendations reduce the cognitive effort to understand complex medical terminology, making health content much easier to access and understand for the general public (6, 7). However, the low barriers for content creation and dissemination on these platforms have also facilitate the proliferation of health misinformation, which can spread at unprecedented speed and scale, outpacing evidence-based corrections and professional guidance (8, 9). Moreover, the rapid growth of AI-generated content has intensified this problem, as artificial intelligence tools makes it easier to produce misleading health information quickly, cheaply, and at scale (10, 11).

The spread of health misinformation on digital platforms poses significant risks to individual and public health outcomes (12). Misinformation can distort risk perceptions, promote harmful behaviors, and erode trust in medical institutions, particularly during public health emergencies such as the COVID-19 pandemic (13, 14). Several studies have consistently documented the high prevalence of inaccurate or unverified health claims across various social media platforms (15, 16), with short videos showing particularly concerning levels of content inaccuracy and poor information quality (17, 18). For instance, assessments of short videos on conditions ranging from myopia to cancer reveal widespread dissemination of non-evidence-based treatments and oversimplified medical advice (19–21). These findings highlight an urgent need to understand how users perceive, process, and evaluate the credibility of health information in the fast-paced, attention-scarce environment of short-video platforms. Therefore, examining how users cognitively process and evaluate health information on short-video platforms is critical for reducing misinformation harms and improving the effectiveness of digital health communication strategies.

Prior studies have identified a range of factors influencing individuals’ susceptibility to health misinformation. Individual differences such as cognitive ability, health literacy, and epistemic beliefs play a critical role in shaping discernment capacity (22–24). Psychological motivations, such as the need for reassurance, control, or social connectedness, may also increase reliance on emotionally appealing but misleading narratives (25). At the content level, characteristics such as narrative coherence, source credibility, and visual production quality further affect perceived trustworthiness (26, 27). Additionally, social environmental cues, such as likes, shares, and comment sentiment serve as heuristic indicators that may override analytical reasoning in information evaluation (28). These findings suggest that misinformation discernment often involves both analytical reasoning and heuristic shortcuts, aligning with the assumptions of dual-process theories such as the Elaboration Likelihood Model (ELM) (29).

Despite these advances, several gaps remain. First, while prior studies have examined health misinformation in online communities or text-based social media, less is known about how users process health information in the audiovisual, fast-paced environment of short videos. Second, most existing models treat influencing factors in isolation or through linear assumptions, overlooking the fact that credibility judgment in short-video contexts is inherently configurational. That’s said, multiple psychological, content, and social environmental cues often interact simultaneously to shape users’ discernment outcomes. Third, although dual-process theories provide a useful framework to understand how users evaluate health information, few studies have translated these cognitive mechanisms into practical and actionable governance strategies for the short-video environment.

To address these gaps, this study adopts fuzzy-set qualitative comparative analysis (fsQCA), which is well-suited for identifying how different combinations of factors jointly lead to accurate or inaccurate detection of misleading health content. Specifically, this research addresses the following two research questions:

RQ1: What are the key factors that influence the users’ ability to identify health misinformation in short videos?

RQ2: How can these key factors be combined into effective configurational pathways to support targeted governance strategies?

## 2 Research design

**Figure 1:**
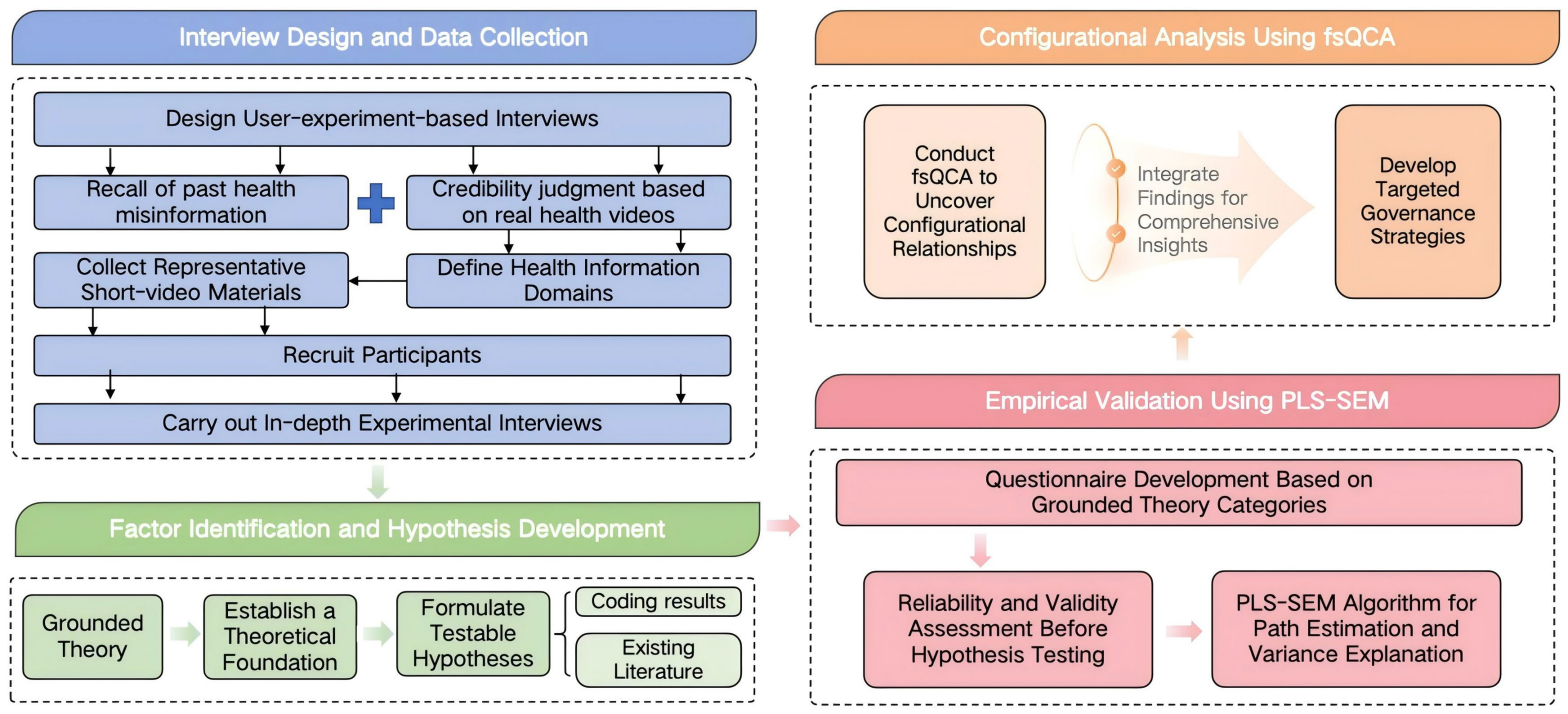
Research workflow.

This study adopted a mixed-methods design combining in-depth interviews, grounded theory, questionnaire surveys, partial least squares structural equation modeling (PLS-SEM), and fuzzy-set qualitative comparative analysis (fsQCA) to comprehensively examine the determinants and mechanisms underlying users’ detection of health misinformation in short videos. The research process included four stages: interview design and data collection, factor identification and hypothesis development, empirical validation using PLS-SEM, and configurational analysis with fsQCA to derive governance implications. The overall workflow is illustrated in Figure 1.

### 2.1 Interview design and data collection

To investigate how users perceive and identify health misinformation in short videos, user-based experimental interviews were conducted to capture participants’ real-time cognitive and evaluative processes. The interview outline is divided into five parts (Table 1).

**Table 1:** Five steps of the interview outline.

Steps	Questions
Personal information and ethical briefing	Explain research objectives, obtaining informed consent Collect basic demographic information
Recollection of recent engagement with short videos	Have you recently watched any short videos related to health? Please provide an example of a health-related short video you have recently watched. Do you find the short video credible, and what criteria do you use to assess its credibility?
On-the-spot video retrieval	What health-related viewpoints did you derive from this short video? How credible do you find this viewpoint? Please elaborate on the reasoning behind your assessment.
Experiment of health misinformation identification	What health-related viewpoints did you derive from the short video? How credible do you find this viewpoint? What criteria or factors did you consider when assessing the credibility of this viewpoint?
Summary and reflection	What criteria do you use to judge the credibility of health-related short videos after the results are revealed? Are there any changes from before?

**Figure 2:**
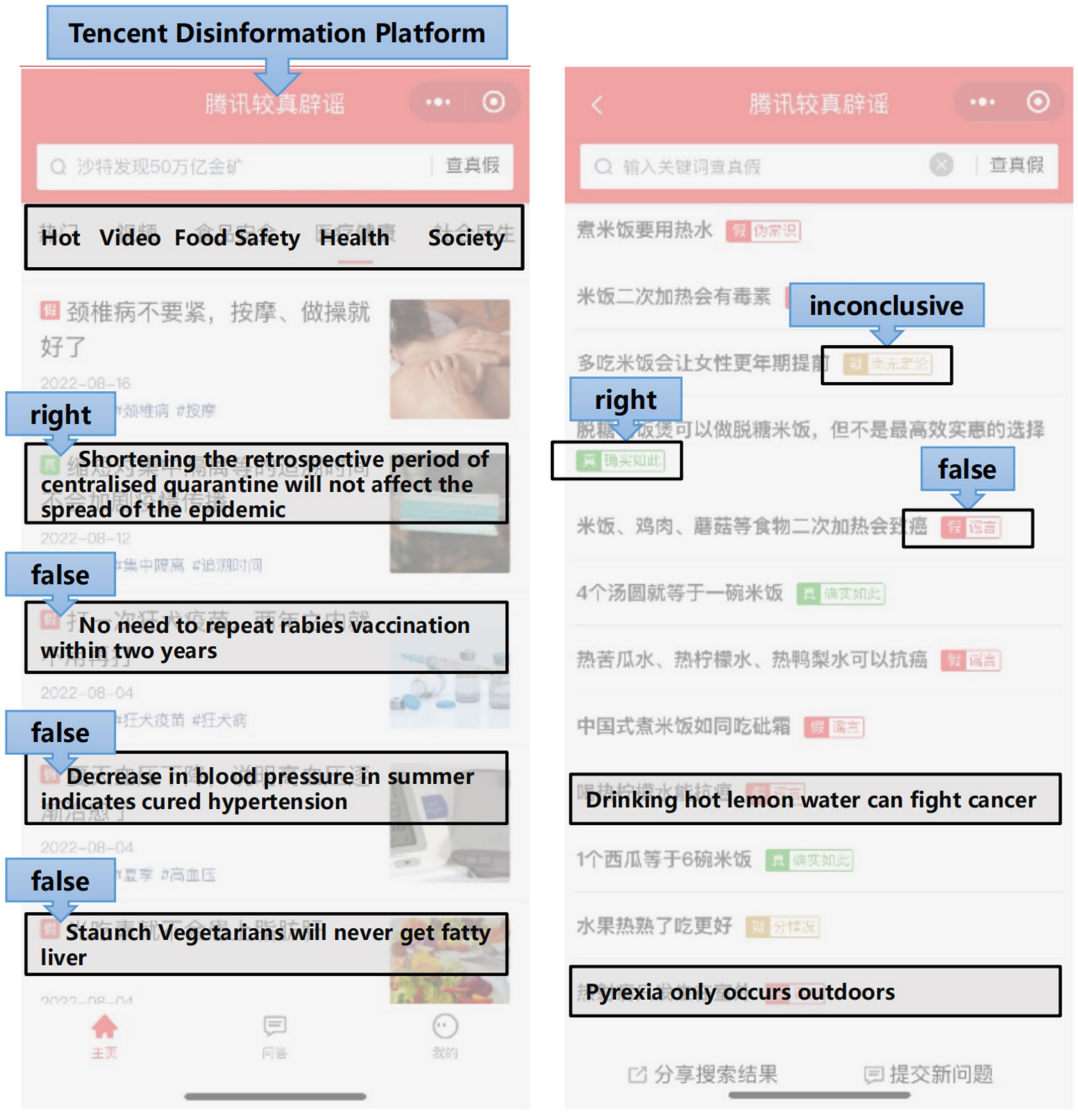
Interfaces of the Tencent Fact Check platform.

In addition to asking participants to recall past encounters with health misinformation, we also presented them with real health-related short videos to serve as the basis for their credibility judgments. This approach helped ground their evaluations in realistic viewing contexts rather than relying solely on memory. To ensure comprehensive coverage of prevalent health topics on short-video platforms, we first identified 7 thematic domains based on the World Health Organization’s public health classifications and existing literature: food safety, wellness practices, public health, health threats, disease prevention, medical environments, and medication usage. Guided by this framework, we systematically selected 50 representative short videos from major Chinese short-video platforms such as Douyin and Bilibili. Each video had been independently verified as either accurate or false by Tencent’s “Jiao Zhen” (Fact-Check) platform (Figure 2), which collaborates with authoritative professional organizations such as the Chinese Medical Association, ensuring a certain level of authority.

Participants were recruited through purposive sampling via online community postings and university networks to ensure diversity in demographic and professional backgrounds. This study fully adhered to ethical guidelines and obtained approval from the Ethics Committee of Beijing University of Chinese Medicine (Approval No.: 2024BZYLL0303). All participants were informed about the purpose and procedures of the study and provided written informed consent prior to participation. In total, 47 participants (22 males, 25 females; aged 18–76 years) took part, including individuals from both healthcare and non-healthcare fields. The interviews ranged from 26 to 91 min, with an average length of 45 min. Notably, all interviews were conducted with the explicit consent of the participants, who were informed about recording the interviews for research purposes. Subsequently, the recorded interviews were meticulously transcribed and organized, resulting in an extensive corpus comprising over 1,50,000 words of rich qualitative data.

### 2.2 Factor identification and hypothesis development

To identify the key determinants influencing users’ ability to detect health misinformation in short videos, a grounded theory approach was employed to analyze 47 interview transcripts using NVivo 14. The analysis followed a three-stage iterative coding procedure comprising open coding, axial coding, and selective coding. Two trained researchers with prior experience in qualitative analysis independently coded the transcripts. Prior to formal coding, they used 3 sample transcripts to align the understanding of code definitions and application rules. Intercoder reliability was assessed using Cohen’s kappa coefficient (*κ* = 0.78), indicating strong agreement among coders. To ensure the robustness of the coding framework, 5 additional transcripts were reserved for theoretical saturation testing. No new categories emerged during this stage, confirming the adequacy of conceptual coverage. Findings from this analysis were synthesized with existing literature to formulate testable hypotheses, establishing a theoretical foundation for subsequent empirical validation.

### 2.3 Empirical validation using PLS-SEM

Building upon the conceptual framework developed in the previous stage, an empirical validation was conducted using PLS-SEM to quantitatively test the hypothesized relationships among key influencing factors of health misinformation discernment in short videos.

The questionnaire was developed based on the main categories and subcategories identified through grounded theory analysis. Each subcategory was operationalized as a measurable variable, and corresponding survey items were designed with reference to validated scales from prior studies. Data was collected between January and March 2024 through online distribution and voluntary participation, ensuring diversity in participant backgrounds.

In total, 284 questionnaires were retrieved, of which 279 were valid, resulting in a 98.24% response validity rate. The gender distribution of respondents was relatively balanced, with 146 males (52.3%) and 133 females (47.7%). Regarding age, our sampling strategy intentionally focused on middle-aged and older adults. This decision was guided by two considerations. First, prior research shows that individuals aged 40 and above are not only frequent consumers of health-related content on short-video platforms but also more vulnerable to misinformation due to lower digital literacy and greater health information needs (30). Second, this age focus aligns with national internet usage trends. According to the 55th Statistical Report on China’s Internet Development (CNNIC), as of December 2024, 51.3% of all internet users in China are aged 40 or above, and 52.5% of adults aged 60 + now use the internet (5). Moreover, for many middle-aged and older adults, short-video platforms such as Douyin serve as a primary gateway to the internet and a major source of health information. Accordingly, our sample comprised 65 young adults (18–39 years), 197 middle-aged adults (40–59 years), and 17 older adults (≥60 years).

All data were analyzed using SmartPLS 4.0. Prior to hypothesis testing, reliability and validity assessments were conducted through Cronbach’s *α*, composite reliability (CR), and average variance extracted (AVE). Subsequently, the PLS-SEM algorithm was employed to estimate the path coefficients, significance levels, and explained variance (*R*^2^) for each dependent variable.

### 2.4 Configurational analysis using fsQCA

While PLS-SEM estimates the effects among latent variables, it relies on the assumption that relationships are linear and symmetric. However, users’ identification of health misinformation in short videos often involves complex and interdependent causal mechanisms that cannot be fully captured by linear models. To address this limitation, we adopted fsQCA as a complementary approach. fsQCA integrates fuzzy set theory and Boolean logic, enabling the examination of asymmetric and configurational relationships among variables. This method allows for the identification of multiple, equally sufficient combinations of factors that lead to the same outcome. By integrating the results of PLS-SEM and fsQCA, we further developed targeted governance strategies to improve users’ misinformation discernment and guide effective platform interventions.

## 3 Theoretical model development

### 3.1 Grounded theory

Grounded theory was employed to construct a theoretical framework that explains how users identify health misinformation in short video contexts. Following the procedures of open coding, axial coding, and selective coding, the study systematically analyzed interview data to extract concepts, categories, and higher-level theoretical constructs.

#### 3.1.1 Open coding

In the first stage, an open coding approach was applied to the raw textual data obtained from in-depth interviews. Each transcript was carefully examined line by line to identify meaningful expressions and recurring patterns. Through iterative comparison and abstraction, a total of 110 initial codes were extracted. To further clarify relationships and reduce redundancy, these initial codes were grouped into 19 conceptual categories (A1–A19) that reflect core aspects of users’ reasoning processes when evaluating health-related short videos. Table 2 presents representative examples illustrating the coding process from initial codes to conceptual categories.

**Table 2:** Examples of open coding: original information, initial codes, and conceptual categories.

Original information	Initial code	Conceptual category
This video cites studies from medical journals, which makes me more likely to trust it.	#Journal citation	A1 Theoretical support
The blood sugar-lowering effect of this drug is supported by relatively clear clinical trial data.	#Experimental data	A2 Data validation
I’ve also seen similar videos elsewhere stating that crossing your legs is harmful to the lumbar spine.	#Cross-source comparison	A3 Source verification
This video is about health policy and law, I think it’s unlikely to be fake.	#Policy and law	A4 Content domain
The video does not clarify whether the frog mentioned is the kind we see in ditches or those sold in markets.	#Lack of detailed description	A5 Insufficient detail
The video consistently explains the side effects of this lipid-lowering drug. It’s quite educational.	#Logical rigor	A6 Well-organized
I always feel like she’s marketing products, as if she’s advertising.	#Advertising intent	A7 Induced tendency
The language used is quite formal and professional.	#Use of medical terminology	A8 Use professional terminology
The video exaggerates the drug’s effect. It does not seem credible or valuable.	#Exaggerated expression	A9 Language style
It’s presented in a documentary format, which makes it feel more trustworthy.	#Documentary-style presentation	A10 Organizational form
It uses AI-generated voiceovers and unclear images. It does not seem very credible.	#AI voiceover	A11 Presentation mode
Whether it’s true or not does not matter. I do not really need it in daily life.	#Irrelevance to self	A12 Practical value
I’m patriotic, so if it says something made in the U. S. harms Chinese people, I tend to believe it.	#Patriotic sentiment	A13 Emotional arousal
This video mentioned the National Medical Products Administration (NMPA), which makes it feel quite professional and authoritative.	#Reference to authority	A14 Authoritative orientation
My neighbor drank alcohol after taking metronidazole and felt sick. The doctor said it was due to the interaction.	#Others’ real-life experiences	A15 Life experience
Just using a strainer can reduce sugar content? That does not sound scientifically reasonable.	#Contrary to common sense	A16 Common sense of science
My niece is a doctor. She’s well-versed in health, so the videos she shares with me feel more trustworthy.	#Shared by relatives	A17 Shared by relatives and friends
Look at the comments! Everyone says it’s fake!	#Negative comments	A18 User comments
This video has so many likes. It must mean that everyone pretty much agrees with it.	#High number of likes	A19 User likes

#### 3.1.2 Axial coding

Axial coding aimed to connect and integrate the conceptual categories identified during open coding, uncovering the underlying logic and forming more abstract and explanatory subcategories. For example, the categories A1 Theoretical support and A2 Data validation were closely related, as users often rely on both to assess the authenticity of health information. These were therefore merged into a higher-level subcategory representing the foundation of information reliability assessment.

Following this procedure, 7 subcategories (B1–B7) were developed. Further synthesis revealed that when users judge the authenticity of short video content, they primarily focus on its verifiability, evidential sufficiency, and internal coherence. Consequently, information reliability and content logic were integrated into the main category of “information quality of short videos.” Applying the same logic, all subcategories were ultimately refined into three main categories (C1–C3), as summarized in Table 3.

**Table 3:** Axial coding results of factors influencing users’ identification of health misinformation in short videos.

Main category	Subcategory	Description
C1 Information quality of short videos	B1 Information reliability	Focus on the authenticity and credibility of the information, including whether there is data proof or theoretical support and whether the source of information can be verified.
	B2 Content logic	Focus on the thematic relevance, accuracy, and consistency of details, as well as the coherence and clarity of content organization.
	B3 Narrative expression	Focus on the way information is conveyed, including linguistic professionalism, tone, and overall communication style.
	B4 Information structure	Focus on the organization and presentation of content, such as voiceover quality, alignment of visuals and text, and overall audiovisual design.
C2 User characteristics of short-video viewers	B5 Psychological needs	Focuses on users’ motivational and emotional dimensions, including their perceived utility of the content and the extent to which it evokes empathy or emotional resonance.
	B6 Cognitive level	Focus on users’ cognitive ability and domain knowledge, such as familiarity with relevant background information, everyday experiences, and scientific literacy.
C3 External environment of short videos	B7 External Influences	Focus on the influence of external environmental factors on user evaluation information, including social media interactions and feedback such as likes, forwards, and comments.

#### 3.1.3 Selective coding

In the selective coding stage, the results of open and axial coding were integrated to develop a conceptual framework explaining how users identify health misinformation in short videos. Three main categories (C1–C3) and their 7 subcategories (B1–B7) were linked to the core concept “misinformation discernment,” which reflects the user’s ability to identify health misinformation. Building on this grounded theoretical structure, the next section translates these categories into measurable constructs and develops a series of hypotheses tested through the PLS-SEM model.

### 3.2 Hypothesis development

**Figure 3:**
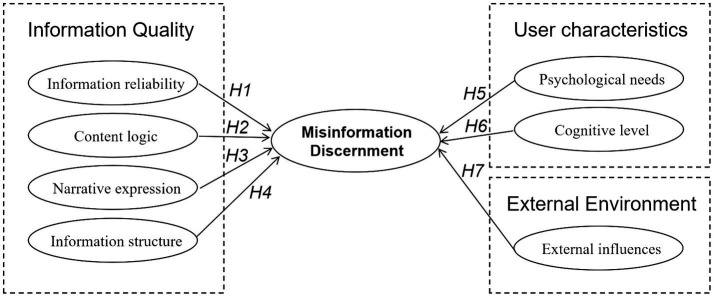
Structural research model.

Based on the results of selective coding, 3 main categories (C1–C3) and 7 subcategories (B1–B7) were identified as the key factors influencing users’ ability to identify health misinformation in short videos. These categories provide the conceptual basis for the theoretical model and the hypotheses proposed below (Figure 3).

#### 3.2.1 Information quality

Information quality refers to features within the message itself, such as verifiability, evidence support, logical consistency, and presentation format. Prior studies have shown that users tend to compare information from multiple sources when judging its authenticity, and that source reliability is a key determinant of credibility evaluation (31, 32). In terms of logical consistency, several studies emphasized that the level of detail and internal coherence are essential indicators of information quality (33, 34), while other study identified logic as a major criterion for evaluating online health information (35). The narrative expression of information, including tone, style, and degree of exaggeration, also affects perceived authenticity. A plain, factual style generally enhances credibility (36), whereas exaggerated language is a common feature of misinformation (37). Moreover, presentation format shapes users’ first impressions. Clear, professional layouts and coherent multimodal presentation (text, images, and visuals) have been shown to improve perceived credibility (38).

Accordingly, the following hypotheses are proposed:

*H1*: Information reliability significantly influences users’ discernment of health misinformation in short videos.

*H2*: Content logic significantly influences users’ discernment of health misinformation in short videos.

*H3*: Narrative expression significantly influences users’ discernment of health misinformation in short videos.

*H4*: Information structure significantly influences users’ discernment of health misinformation in short videos.

#### 3.2.2 User characteristics

User characteristics refer to individual attributes such as cognitive ability, domain knowledge, and psychological needs that shape how information is processed. Previous research indicates that perceived usefulness, relevance, and interest strongly influence users’ overall credibility judgments (39). Cognitive ability, emotional state, and personality traits further affect health information comprehension and evaluation, as users with higher literacy and analytical capacity tend to detect misleading cues more effectively (40, 41).

Based on these findings, the following hypotheses are proposed:

*H5*: Psychological needs significantly influences users’ discernment of health misinformation in short videos.

*H6*: Cognitive level significantly influences users’ discernment of health misinformation in short videos.

#### 3.2.3 External environment

External environment refers to platform-level cues such as likes, comments, shares, and peer recommendations. These cues influence users’ perception and information evaluation processes, indirectly shaping their ability to recognize misinformation, particularly when information quality or personal expertise is insufficient. Empirical studies have shown that social support enhances users’ capacity to discern reliable health information, while negative comments can reduce trust in health-related posts (42, 43). Conversely, endorsement cues like large numbers of likes or shares may increase the perceived credibility of false content (44).

Accordingly, the following hypothesis is proposed:

*H7*: External influences significantly influences users’ discernment of health misinformation in short videos.

## 4 Empirical analysis

### 4.1 Questionnaire design

Based on the research model developed in the previous section, the questionnaire was designed to measure the factors influencing users’ discernment of health misinformation in short videos. Consistent with prior research on online information credibility and health misinformation processing, all questionnaire items were measured using a 7-point Likert scale (1 = strongly disagree; 7 = strongly agree). A total of 22 items were developed across the 8 variables, and the complete list of measurement items is presented in Table 4. These measurement items formed the foundation for the subsequent analyses, including reliability and validity assessment, PLS-SEM structural modeling, and fsQCA configurational analysis.

**Table 4:** Measurement items for the survey questionnaire.

Category	Variable	Item label	Measurement item (7-point Likert scale)
Information quality	Information reliability (IR)	Q1	Health short videos with clear professional evidence are more credible to me.
		Q2	Health short videos supported by data are more credible to me.
		Q3	Health short videos with verifiable sources are more credible to me.
	Content logic (CL)	Q4	The topic of a health short video affects how credible I find it.
		Q5	Health short videos with objective and detailed content are more credible to me.
		Q6	Health short videos that are well-organized and logical are more credible to me.
	Narrative expression (NE)	Q7	Health short videos without advertising or persuasive intent are more credible to me.
		Q8	Health short videos using professional and fluent language are more credible to me.
		Q9	Health short videos with a professional tone or style are more credible to me.
	Information structure (IS)	Q10	The presentation format of health short videos (e.g., news style, animation, documentary) affects my credibility judgement.
		Q11	Health short videos with clear visuals and smooth playback are more credible to me.
User characteristics	Psychological needs (PN)	Q12	Health short videos that help solve my practical problems are more credible to me.
		Q13	Health short videos that evoke empathy are more credible to me.
		Q14	Health short videos from authoritative platforms or publishers are more credible to me.
	Cognitive level (CG)	Q15	Health short videos on topics I am familiar with are more credible to me.
		Q16	Health short videos consistent with common sense are more credible to me.
External environment	External influences (EI)	Q17	Health short videos shared by my friends or family are more credible to me.
		Q18	User comments on health short videos affect my credibility judgement.
		Q19	Health short videos with higher public engagement (e.g., likes, shares, views) are more credible to me.
Dependent variable	Misinformation discernment (MD)	Q20	I can apply critical thinking to judge the authenticity of health short videos.
		Q21	I can accurately understand the meaning conveyed in health short videos.
		Q22	I can identify misleading or inaccurate statements in health short videos.

### 4.2 PLS-SEM results

#### 4.2.1 Reliability and validity testing

Reliability and validity tests were conducted using SmartPLS 4.0. The analysis showed that the IR variable exhibited low internal consistency (Cronbach’s *α* = 0.656, CR = 0.674) when item Q3 was included, both below the recommended threshold of 0.7. After removing Q3, the reliability metrics improved considerably (see Table 5). After the revision, all variables demonstrated satisfactory internal consistency (Cronbach’s α > 0.7, CR > 0.7). Convergent validity was also supported, as all constructs achieved an average variance extracted (AVE) > 0.5 and CR > 0.7.

**Table 5:** Reliability and validity testing.

Variable	Item label	Standardized loading	Cronbach’s α	AVE	CR
IR	Q1	0.911	0.715	0.776	0.744
	Q2	0.850			
CL	Q4	0.701	0.779	0.700	0.794
	Q5	0.897			
	Q6	0.898			
NE	Q7	0.868	0.859	0.781	0.861
	Q8	0.905			
	Q9	0.877			
IS	Q10	0.926	0.785	0.822	0.807
	Q11	0.887			
PN	Q12	0.869	0.826	0.741	0.829
	Q13	0.852			
	Q14	0.861			
CG	Q15	0.898	0.747	0.798	0.748
	Q16	0.888			
EI	Q17	0.851	0.885	0.814	0.895
	Q18	0.928			
	Q19	0.925			
MD	Q20	0.837	0.875	0.800	0.889
	Q21	0.931			
	Q22	0.912			

#### 4.2.2 Hypothesis testing

**Figure 4:**
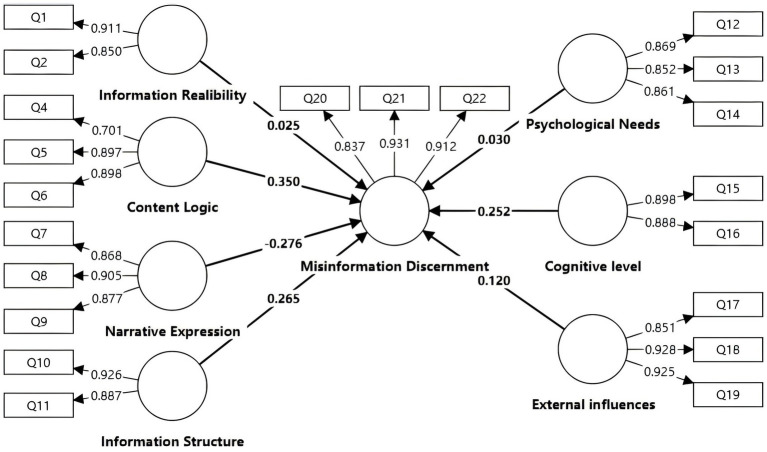
Research model of hypothesis testing.

Data analysis was conducted using partial least squares structural equation modeling (PLS-SEM) in SmartPLS 4.0 to evaluate the hypothesized relationships, influence pathways, and effects of distinct paths on endogenous variables. Before estimating the structural model, multicollinearity diagnostics were conducted. All variance inflation factor (VIF) values were below the recommended cutoffs (VIF < 5), indicating no multicollinearity concerns among predictor variables. The structural model (Figure 4) demonstrated moderate explanatory power for identification capability (*R*^2^ = 0.478), supporting the feasibility of subsequent path analysis. Global fit indices were acceptable (SRMR = 0.066; NFI = 0.751). Bootstrapping (1,000 resamples) was used to obtain standard errors, *t*-values, and 95% confidence intervals for all path coefficients. Effect sizes (f^2^) were computed to assess the substantive impact of each predictor. Results indicated that hypotheses H2, H3, H4, H6, and H7 were statistically supported (*p* < 0.05); H1 and H5 were not supported (see Table 6 for details).

**Table 6:** Hypothesis testing results of the research model.

Hypothesis	Path	Path coefficient	*p*-value	f^2^	VIF	Result
H1	IR → MD	0.025	0.381	0.001	2.262	Not support
H2	CL → MD	0.350	0.015^*^	0.057	4.077	Support
H3	NE → MD	−0.276	0.017^*^	0.032	4.610	Support
H4	IS→MD	0.265	0.006^**^	0.041	3.315	Support
H5	PN → MD	0.030	0.395	0.000	4.416	Not support
H6	CG → MD	0.252	0.013^*^	0.032	3.786	Support
H7	EI → MD	0.120	0.044^*^	0.024	1.916	Support

*p < 0.05, **p < 0.01, ***p < 0.001.

### 4.3 FsQCA results

#### 4.3.1 Data calibration

Following standard fsQCA procedures, we first computed the average score of all items under each construct to obtain composite indicators by fsQCA 3.0. These indicators were then calibrated into fuzzy membership scores. Consistent with prior fsQCA studies in public health and information behavior, we used the 75th, 50th, and 25th percentiles of the sample distribution as the three qualitative anchors representing full membership, crossover point, and full non-membership, respectively (45, 46). To assess robustness, we conducted a calibration sensitivity test by shifting the anchors (0.7, 0.5, 0.3). The resulting configurations remained stable in terms of consistency, coverage, and core conditions, indicating that our findings are not overly dependent on a specific threshold choice.

#### 4.3.2 Necessary conditions

Necessary conditions were evaluated using the conventional consistency benchmark of 0.90. As shown in Table 7, no single antecedent reached this threshold for the outcome of high identification capability. All consistency scores were below 0.90, indicating that none of the conditions can be considered necessary for achieving the outcome. This suggests that misinformation discernment is better understood through configurations of multiple conditions rather than single-variable explanations.

**Table 7:** Results of the necessity condition test.

Antecedent variables	Consistency	Coverage
CL	0.803	0.736
~CL	0.327	0.281
NE	0.805	0.712
~NE	0.337	0.300
IS	0.819	0.720
~IS	0.311	0.279
CG	0.807	0.774
~CG	0.351	0.290
EI	0.740	0.692
~EI	0.372	0.314

#### 4.3.3 Configuration analysis

Configuration analysis was conducted by constructing a truth table and generating the complex, intermediate, and parsimonious solutions. Among them, the intermediate solution is generally regarded as the most informative. According to Fiss’s reporting guidelines (47), conditions that appear in both the intermediate and parsimonious solutions are considered core conditions (indicated by “●”), whereas conditions that appear only in the intermediate solution are treated as peripheral conditions (indicated by “·”). Absence of a condition in a configuration is left blank to indicate irrelevance for that pathway. Based on these principles, further configurational analysis was conducted using fsQCA 3.0, and the results are summarized in Table 8.

**Table 8:** Results of configuration analysis.

Antecedent variables	Solution 1	Solution 2	Solution 3
CL	●	·	
NE	●	●	●
IS	●	●	●
CG	●		·
EI		●	●
Consistency	0.852	0.872	0.875
Raw coverage	0.704	0.619	0.622
Unique coverage	0.098	0.013	0.016
Overall consistency	0.840		
Overall coverage	0.732		

“●” denotes core conditions,“·” denotes peripheral conditions.

According to Table 8, The model yields three sufficient configurations, with consistency values of 0.852, 0.872, and 0.875, respectively. The raw coverage for each configuration ranges from 0.619 to 0.704, and the unique coverage from 0.013 to 0.098, indicating that each configuration explains a meaningful share of the outcome. Overall, the model demonstrates an overall consistency of 0.840 (greater than the recommended 0.80 threshold), suggesting a high degree of reliability. The overall coverage reaches 0.732, exceeding the commonly accepted 0.50 benchmark and indicating a strong explanatory power of the combined configurations.

By grouping configurations that share the same core conditions, three distinct types of causal patterns can be identified:

1. Configuration S1 is characterized by four core conditions: content logic (CL), narrative expression (NE), information structure (IS), and cognitive level (CG), indicating a pathway driven by strong analytical and information-processing mechanisms. This combination suggests that when users possess a relatively high level of cognitive ability and simultaneously attach importance to information quality (e.g., logical content, coherent narratives, and well-organized structures), they tend to invest more time and effort in examining short-video content. Users in this configuration rely primarily on internal cues, evaluating the underlying logic, clarity of expression, and structural completeness of information to judge its authenticity. External Influences (EI) is not required in this pathway, highlighting a predominantly rational and cognition-centered mechanism for discerning misinformation.
2. In Configuration S2, narrative expression (NE), information structure (IS), and external influences (EI) emerge as core conditions, while content logic (CL) plays a peripheral role. This pattern shows that although logical consistency has some effect on the identification process, it is not as influential as the other three factors. This configuration typically reflects users with lower or moderate cognitive capacity, who may not systematically analyze the logical structure of the content. Instead, their judgments are more strongly shaped by professionally presented narratives, visually or structurally appealing formats, and positive external cues, such as endorsements, comments, or social validation. As a result, users tend to rely on external and stylistic features of the video rather than its internal logical rigor, using these visible cues as heuristics to assess credibility.
3. Configuration S3 also identifies narrative expression (NE), information structure (IS), and external influence (EI) as core conditions, but with cognitive level (CG) functioning as a peripheral condition. This indicates that while users possess some degree of health information literacy, they still primarily depend on peripheral cues such as narrative style, presentation structure, and social feedback to judge authenticity. Even when the content lacks strong logical reasoning or contains inconsistencies, these users may overlook such deficiencies and place greater trust in videos that appear professionally narrated, visually organized, or socially endorsed.

## 5 Discussion

### 5.1 Main findings

The findings of this study demonstrate that health misinformation discernment in short videos is co-determined by three dimensions: information quality, user characteristics, and external environment. These factors do not operate in isolation but exert synergistic effects through distinct configuration paths. This suggests that effective governance must move beyond single-dimensional limitations and establish a multi-dimensional collaborative model for managing health misinformation in short videos.

1. Information quality dimensions have distinct effects on misinformation identification. Content logic (H2) and information structure (H4) significantly enhance users’ identification ability, whereas narrative expression (H3) has a significant negative effect. Information reliability (H1) is not significant. These results suggest that improving content organization and reducing misleading narrative practices should be central to governance efforts. Specifically, content logic is the strongest predictor, suggesting that users evaluate the authenticity of health information primarily by assessing logical coherence (48). Information structure also plays a meaningful role, which aligns with the rapid, visually oriented processing typical of short-video platforms (49). Conversely, narrative expression negatively impacts discernment, suggesting that professional or polished narrative styles may lower users’ vigilance in the short-video context. Because misleading health content often adopts the same fluent and neutral tone, users may treat these cues as signals of credibility, making it harder for them to discern misinformation (50).
2. Within user characteristics, cognitive level (H6) significantly improves discernment. This underscores the importance of strengthening users’ analytical capabilities in misinformation governance. Prior research similarly shows that individuals who engage in more reflective reasoning are better at detecting inaccuracies, while reliance on intuitive thinking increases susceptibility to misinformation (51, 52). Notably, psychological needs (H7) did not show a significant effect. This suggests that while personal relevance or emotional resonance may increase a user’s willingness to believe information, this motivational bias does not directly translate into an enhanced ability to accurately identify its veracity, emphasizing the primacy of rational cognition over psychological desires.
3. External influences (H8) has a significant impact, highlighting the importance of purifying the information ecosystem. Identifying health misinformation is a highly social and contextual process. In highly interactive social media platforms like short videos, users’ judgments are often influenced by a group identification effect (53), in which they unconsciously refer to or rely on the opinions and behaviors of others, including friends’ shares, user comments, and like counts, when making decisions (54).
4. Configuration analysis identifies 5 effective antecedent configurations, which can be grouped into 3 pathways aligned with the Elaboration Likelihood Model (ELM). The Analytical Central Route relies on content logic, narrative expression, information structure, and cognitive level as core conditions, representing high-cognition users engaging in deep analysis. The Content-assisted Peripheral Route relies on narrative expression, information structure, and external influences, with content logic as a supplementary condition, reflecting low-cognition users who depend on superficial cues such as professional language or engagement metrics. The Cognition-assisted Peripheral Route also focuses on narrative expression, information structure, and external influence, but is supplemented by cognitive level, representing medium-cognition users who possess some analytical capacity but still favor peripheral cues. These results reveal the diversity of user information processing modes, necessitating correspondingly differentiated governance strategies.

### 5.2 Governance implications

**Figure 5:**
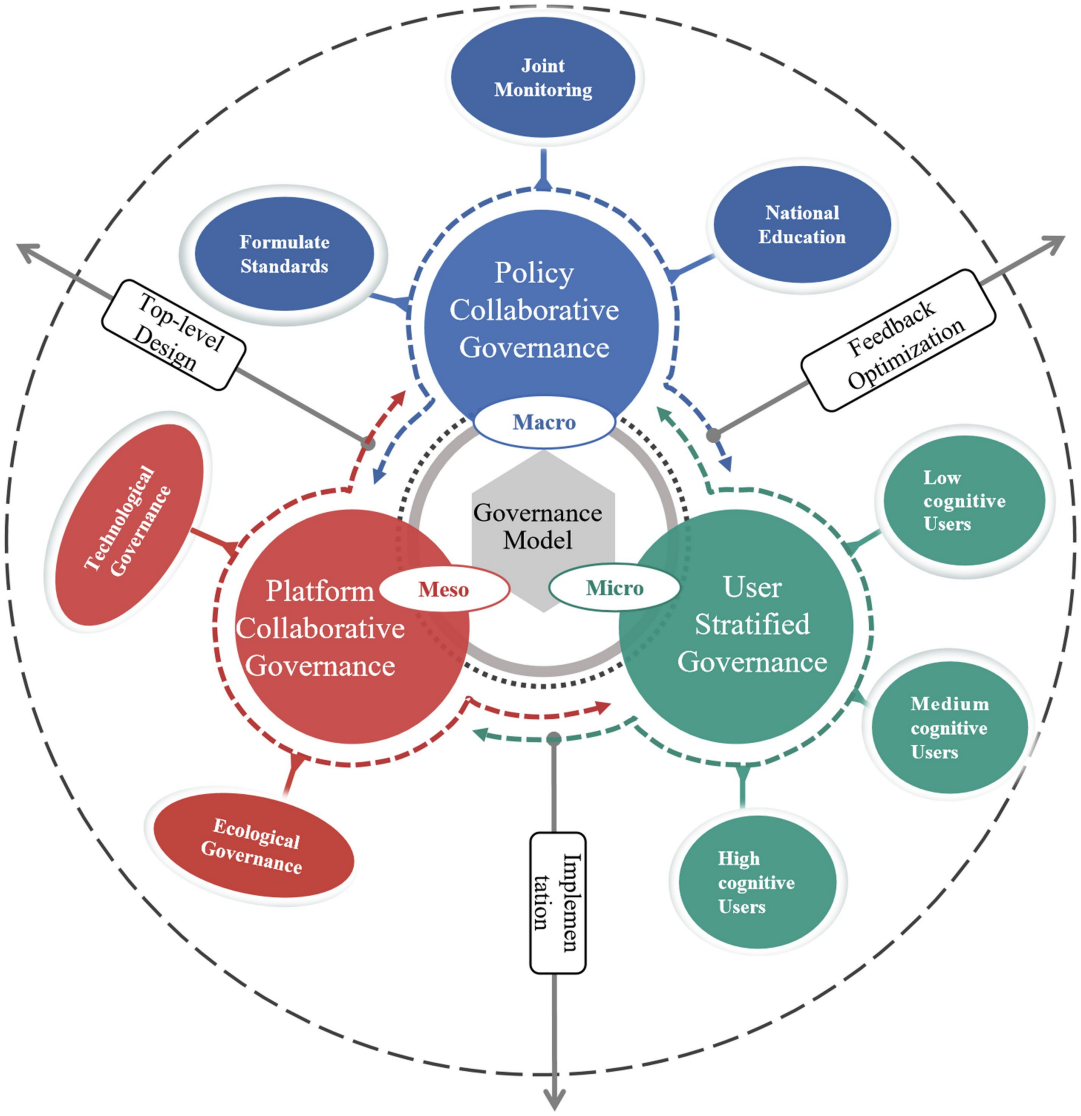
A multi-level governance framework of health misinformation in short videos.

Based on the influencing-factor model and the configuration results, this study develops a multi-level governance framework for addressing health misinformation in short videos (Figure 5). The framework illustrates how users’ misinformation discernment is shaped by the joint effects of information quality, user characteristics, and the external environment. We organize governance pathways into 3 interconnected layers: micro (user-stratified governance), meso (platform collaborative governance), and macro (policy collaborative governance). These layers interact through feedback and implementation loops, highlighting that effective governance requires both differentiated user-level interventions and coordinated platform- and policy-level actions.

#### 5.2.1 Micro level: differentiated governance for heterogeneous user groups

Configuration analysis reveals three primary user archetypes: high-cognition, medium-cognition, and low-cognition users, necessitating tailored governance strategies.

For high-cognition users, who typically engage the central route of analytical processing, governance should focus on deep empowerment. This involves providing powerful analytical tools and ensuring access to high-quality information sources to support their rational judgment. For instance, interactive verification tools embedded within platforms, potentially leveraging existing feature lists of pseudo-health information, could allow users to compare content against known misinformation characteristics swiftly (55). For medium-cognition users, who employ a cognition-assisted peripheral path, governance should aim to optimize their information environment and guide their cognitive abilities toward more analytical processing. Establishing personalized education systems that deliver customized information literacy training can strengthen their analytical engagement and reduce reliance on peripheral cues. For low-cognition users, who depend heavily on peripheral information, governance should focus on creating a simplified and safer decision environment. This can be achieved by algorithmically prioritizing the display of high-quality information to these users, adding credibility prompts to highly disseminated videos, and implementing intuitive credibility rating systems (e.g., using color-coded labels) to translate complex quality judgments into easily understandable decision heuristics.

#### 5.2.2 Meso level: platform-centered techno-ecological governance

At the meso level, build an orchestrated techno-ecological governance system with platforms at its core. The findings underscore that information quality, particularly content logicality and structure, is central to users’ identification abilities. Therefore, platforms should take a leading role by developing and deploying advanced algorithms for detecting health misinformation in short videos. This involves conducting multi-modal analysis of logical coherence, evidence support, and narrative plausibility to automatically flag high-risk content. Simultaneously, it is crucial to build recommendation models based on multi-dimensional quality metrics, giving preferential exposure to high-quality health content to enhance the overall information environment at its source. Furthermore, the comment section, as a significant component of the external environment, exerts a notable influence on user judgment. Platforms must, therefore, actively cultivate a high-quality comment ecology. Optimizing comment sorting algorithms to prioritize “quality comments” characterized by rational discussion and authoritative citations is essential. Encouraging contributions from users with professional backgrounds can foster a constructive reference environment, particularly benefiting low- and medium-cognition users who are more susceptible to external influences.

#### 5.2.3 Macro level: targeted and collaborative governance mechanisms

At the macro level, the focus should be on constructing a targeted and collaborative governance framework. The significant impact of the external environment highlights the importance of macro-level policies. Consequently, it is recommended that relevant authorities formulate explicit industry standards for the quality of health-related short videos, specifying normative requirements for source disclosure, evidence levels, and review protocols. Strengthening accountability mechanisms is equally important, clarifying the responsibilities of content creators, platforms, and sharers, and imposing stricter penalties for the dissemination of harmful health misinformation. In addition, a dynamic cross-sector monitoring and response system is needed. Regular coordination among public health authorities, cyberspace regulators, industry associations, and platform enterprises can enable real-time tracking of the spread of high-risk content and more efficient intervention. Finally, given the strong effect of cognitive level on identification capability, integrating health information literacy into national education initiatives is crucial. Public campaigns that build critical thinking about health information, combined with efforts by authoritative institutions to provide accurate and accessible health knowledge, can help reduce the spread of health misinformation.

### 5.3 Limitations

While this study developed a relatively comprehensive theoretical model using a mixed-methods approach, several limitations should be acknowledged. First, the model’s explanatory power (*R*^2^ = 0.478) indicates that the model may not capture all factors that influence users’ discernment of health misinformation in short videos, such as platform algorithm features or cultural differences. Future research could incorporate these variables to explore their impact more thoroughly. Second, as the sample was drawn from the context of Chinese, the findings may be generalizable to other countries with similar cultural backgrounds. However, its applicability to other countries or regions that differ significantly from China requires further empirical examination. Future studies could expand the sample to include participants from diverse cultural settings to enable cross-cultural comparisons. Finally, the reliance on self-reported data from surveys and interviews introduces the potential for subjective bias. Subsequent research would benefit from incorporating behavioral log data or other objective measures to validate and refine the proposed theoretical model.

## 6 Conclusion

Short-video platforms have become a major source of health information, yet their unregulated content environment increases users’ exposure to misinformation. This study identifies key factors and configurational pathways that shape how users discern health misinformation. Quantitative analysis shows that content logic, information structure, cognitive level, and external influence enhance the discernment, while highly stylized narrative expression may undermine it. Complementing this, fsQCA reveals 3 distinct processing pathways: the central route of analytical processing, the content-assisted peripheral route, and the cognition-assisted peripheral route, demonstrating that effective judgment arises from multiple combinations of factors rather than any single predictor. The combined application of these two methods can provide practical insights for optimizing information services on short video platforms, enhancing the construction of information resources, and improving users’ capacity to identify health-related misinformation.

## Data Availability

The raw data supporting the conclusions of this article will be made available by the authors, without undue reservation.
